# Identification and Analysis of Senescence-Related Genes in Head and Neck Squamous Cell Carcinoma by a Comprehensive Bioinformatics Approach

**DOI:** 10.1155/2022/4007469

**Published:** 2022-10-17

**Authors:** Lin Deng, Jinglin Mi, Xiaolan Ruan, Guozhen Zhang, Yufei Pan, Rensheng Wang

**Affiliations:** ^1^Department of Oncology, The First Affiliated Hospital of Guangxi Medical University, Nanning 530021, China; ^2^Department of Radiation Oncology, Nanxishan Hospital of Guangxi Zhuang Autonomous Region, Guilin 541004, China

## Abstract

Head and neck cancer is the sixth most frequent cancer all over the world, with the majority of subtypes of head and neck squamous cell carcinoma (HNSCC). Cellular senescence-associated genes have been confirmed to play a critical role in cancer and have the potential to be prognostic biomarkers for cancer. Clinical information of HNSCC samples and expression data were acquired from public databases. Expression profiles of genes related to cellular senescence were used to identify molecular subtypes by consensus clustering. To screen differentially expressed genes (DEGs) between different subtypes, differential analysis was performed. We used the univariate Cox regression to identify prognostic DEGs and performed least absolute shrinkage and selection operator (LASSO) to optimize and construct a prognostic model. CIBERSORT, ESTIMATE, and TIDE tools were applied to estimate immune characteristics. Four molecular subtypes were established based on cellular senescence-associated genes. Differential prognosis was observed among different subtypes with C4 having the longest overall survival and C1 having the worst prognosis. C4 subtype also showed the highest immune infiltration. We screened a total of eight cellular senescence prognosis-related genes and established a cellular senescence-related signature score (CSRS.Score) that could stratify samples into high-CSRS.Score and low-CSRS.Score groups. The high-CSRS.Score group had worse prognosis, lower immune infiltration, and lower response to immunotherapy. We further improved the prognostic model and survival prediction by combining CSRS.Score with clinicopathological features using a decision tree model, which had high predictive accuracy and survival prediction. This study demonstrated an important role of cellular senescence in HNSCC. The identified eight cellular senescence-associated genes have the potential to provide ideas for adjuvant treatment and personalized treatment of HNSCC patients.

## 1. Introduction

Head and neck cancer (HNC) is the sixth most frequently diagnosed cancer type that causes 500,000 affected individuals per year worldwide [1]. Head and neck squamous cell carcinoma (HNSCC) accounts for the majority HNC patients, and more than half of the patients with HNSCC are initially diagnosed with locally advanced disease [2, 3]. Lymph node (LN) metastasis is a negative signal of head and neck cancer prognosis. However, it is challengeable to identify metastatic LN within the fibroadipose tissue [4]. The prognosis for HNSCC remains poor even with the use of combination therapy including surgery, radiation, chemotherapy, and immunotherapy [5]. Although no tumor LN is detected from clinical and radiographic estimation, there is still a high possibility over than 30% to observe nodal metastasis in the surgery [6]. Therefore, there is an urgent need to provide effective biomarkers for early diagnosis, personalized treatment, and prognostic evaluation.

Senescence is a nearly unavoidable feature in all creatures, which is marked by a descending function of multiple cells and tissues. In spite of that degeneration is the most common age-related phenotype, aging allows to generate gain-of-function changes that lead to abnormal cell proliferation [7]. Moreover, these changes can result in genomic instability that enable to provide an advantage for the abnormal cells in proliferation, migration, and escape from immune surveillance [8]. Obviously, these phenotypes are the hallmarks of malignant cancers. Senescence plays a two-sided role in cancer development, preventing tumorigenesis by cell growth arrest in precancerous cells, but also facilitating malignant transformation of adjacent cells through protumorigenic drivers [9, 10]. Senescent cells can alter epigenetic modifications in neighboring cells by releasing senescence-associated signals [11–13]. A number of genes have been demonstrated to regulate senescence in cancer cells, such as p53 [14], Raf1 [14], MAP2K6/p38 [15], and PTEN [16]. Therapy-induced senescence has been observed in cancer cells after radiotherapy or chemotherapy [17]. When exposed to various conventional and targeted anticancer drugs, tumor cell senescence is induced, resulting in a positive effect on patient treatment [18, 19]. Thus, senescence is considered as a therapeutic target for clinical cancer treatment [20].

Senescence-associated genes also have the great potential to predict cancer prognosis. Althubiti et al. identified 10 plasma membrane-associated proteins expressed in senescent cells to be prognostic biomarkers especially in breast cancer [21]. Coppola et al. discovered a series of senescence-associated genes that correlated with age, overall survival, and grade of glioblastoma [22]. Yang et al. identified seven age-related genes by analyzing the expression profiles of HNSCC and adjacent cancer samples [23]. The risk score based on the seven age-related genes was significantly related to prognosis and immune response. But none of the studies have explored a molecular subtyping system based on senescence-associated genes in HNSCC. Senescence-associated molecular subtypes may help to further understand the role of cellular senescence in the tumor progression.

Therefore, in this study, we used cellular senescence-associated genes to identify molecular subtypes. Differential pathways and immune features were observed among different subtypes. Differential expressed genes (DEGs) were screened between different subtypes, and least absolute shrinkage and selection operator (LASSO) regression analysis was used to develop a cellular senescence-related signature scoring (CSRS) system. The CSRS system could define CSRS.Score for each HNSCC sample and classify them into high-CSRS.Score and low-CSRS.Score groups. Importantly, CSRS.Score had the potential to guide immunotherapy and chemotherapy for HNSCC patients. A decision tree and a nomogram based on CSRS.Score were constructed to more accurately predict prognosis than CSRS.Score only.

## 2. Materials and Methods

### 2.1. Data Source and Preprocessing

From The Cancer Genome Atlas (TCGA) database (named as TCGA cohort), we downloaded RNA-seq data of HNSCC samples and removed samples that did not have survival time, clinical follow-up information, or status of patients' survival. Ensembl ID was transformed into gene symbol. The median value of gene expression was selected for the genes with multiple gene symbols. GSE65858 and GSE41613 cohorts including gene expression profiles of HNSCC samples were obtained from Gene Expression Omnibus (GEO) database and were used as validation cohorts. We downloaded the annotation information of the corresponding microarray platform and mapped the probes to genes based on the annotation information to remove the probes that match one probe to multiple genes. If certain number probes matched to one gene, the median value was taken as the expression value of that gene. Finally, 499, 253, and 97 samples were remained in TCGA, GSE65858, and GSE41613 cohorts, respectively.

From CellAge database (https://genomics.senescence.info/cells/), we obtained 279 cellular senescence-associated genes.

### 2.2. Molecular Typing Based on Cellular Senescence-Associated Genes

We next constructed a consistency matrix by ConsensusClusterPlus to cluster HNSCC samples [24]. The expression data of genes associated with cellular senescence was used as a basis to obtain the molecular subtypes of the samples. “Pam” algorithm and “1 - Spearman correlation” were determined as a metric distance to perform 500 bootstraps, with each bootstrap consisting of 80% patients in the training set. Cluster numbers were set from 2 to 10, and the optimal cluster was determined by cumulative distribution function (CDF) and consensus matrix. Finally, the confirmed clusters were the molecular subtypes.

### 2.3. Construction of a CSRS.Score Scoring System

We identified differentially expressed cellular senescence genes between subtypes using limma R package [25] and selected prognostically significant differentially expressed genes through the univariate Cox regression analysis (*P* < 0.05). LASSO regression using the glmnet R package [26] and stepwise Akaike information criterion (stepAIC) [27] were performed to compress and reduce the differential genes to obtain prognostic genes associated with cellular senescence. The CSRS.Score for each sample was shown as follows: CSRS.Score = *Σ* *βi* × Expi, where Expi indicates the gene expression level of prognostic genes and *βi* is Cox regression coefficients of the corresponding genes. CSRS.Score was normalized using *z*-score, and the threshold “0” was determined to classify samples into low-risk and high-risk groups. The Kaplan-Meier (KM) survival analysis was conducted to assess the overall survival of different molecular subtypes. Significant differences were determined using the log-rank test.

### 2.4. Assessment of Immune Infiltration

CIBERSORT algorithm was employed to estimate the proportion of 22 immune cell types [28]. ESTIMATE algorithm was used to calculate stromal score and immune score for evaluating stromal and immune infiltration [29].

### 2.5. Prediction of Immunotherapy Responsiveness

We used the TIDE algorithm to validate predicted treatment responsiveness. The TIDE algorithm is a computational method for predicting immune checkpoint blockade responsiveness using gene expression profiles [30]. TIDE can use gene expression information to predict cancer sensitivity to immune checkpoint therapy. The TIDE algorithm evaluates three immunosuppressive cell types that limit T cell infiltration in tumors, including M2 tumor-associated macrophages (TAMs), tumor-associated fibroblasts (CAFs), and myeloid-derived suppressor cells (MDSCs). The dysfunction score of tumor-infiltrating cytotoxic T lymphocytes (CTLs) (T cell dysfunction) and the exclusion score of CTLs by immunosuppressive factors (T cell exclusion) can be calculated by TIDE analysis.

### 2.6. Gene Set Enrichment Analysis (GSEA)

GSEA allows to calculate the enrichment score of a gene set for annotating biological function [31]. We used GSEA for Kyoto Encyclopedia of Genes and Genomes (KEGG) pathway analysis in different molecular subtypes. Gene sets of KEGG pathways were accessed from the Molecular Signature Database (MSigDB) [32]. The enrichment scores of aneuploidy, homologous recombination defect number of segments, and fraction altered were calculated using GSEA.

### 2.7. Statistical Analysis

R software (v4.1) was applied to conduct all statistical analysis. The Kruskal-Wallis test was performed in testing the significance among four subtypes. Between high- and low-risk groups, the Wilcoxon test was performed to test the significance. Log-rank test was conducted in the Cox regression analysis and survival analysis. ANOVA was conducted in comparing different groups containing multiple subgroups. *P* < 0.05 was considered as significant.

## 3. Results

### 3.1. Molecular Typing Based on Cellular Senescence-Associated Genes

**Figure 1:**
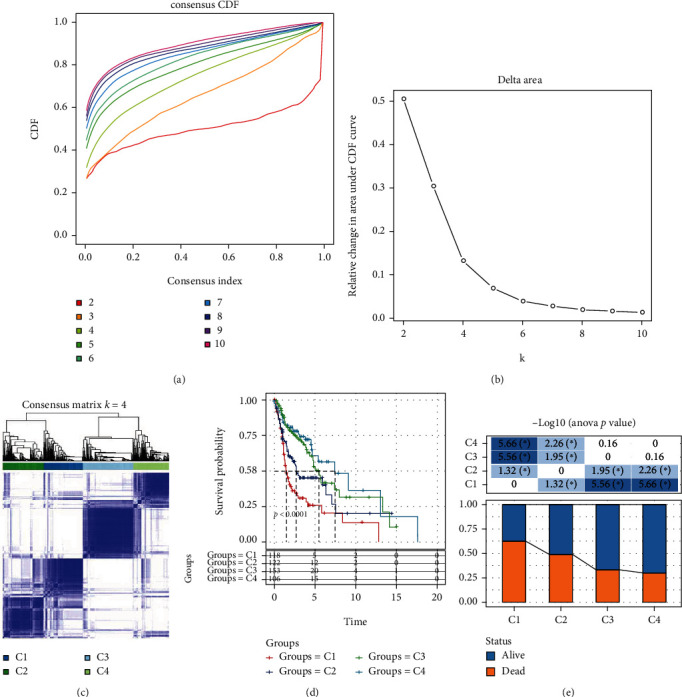
The TCGA cohort molecular typing based on cellular senescence-associated genes. (a) CDF curve for TCGA cohort samples. (b) CDF delta area curve for TCGA cohort samples. The vertical axis represents the relative change in area under CDF curve, and the horizontal axis represents the category number *k*. (c) At consensus *k* = 4, heat map of sample clustering. (d) KM curve of the four subtypes. (e) Survival status differences in different subtypes. ^∗^*P* < 0.05.

First, we extracted the expression of cellular senescence-associated genes from TCGA cohort. Then, the univariate Cox regression analysis was performed, and we obtained 28 genes associated with prognosis (Table S1) (*P* < 0.01). Then, based on the expression data of 28 prognosis-related cellular senescence-associated genes, we clustered 499 HNSCC samples into four clusters (molecular subtypes) through the determination of the CDF and the CDF delta area (Figures 1(a) and 1(b)). The consensus matrix showed that four clusters were independently distributed for most samples (Figure 1(c)). KM survival curves displayed that four molecular subtypes had significant differences of overall survival (*P* < 0.01, Figure 1(d)), with C4 having the most favorable prognosis and C1 having the highest proportion of dead samples (*P* < 0.05, Figure 1(e)). In addition, C1 also had a higher proportion of advanced stages of T stage, N stage, and AJCC stage (Figure S1).

### 3.2. Genomic Characteristics and Enriched Pathways of Molecular Subtypes

**Figure 2:**
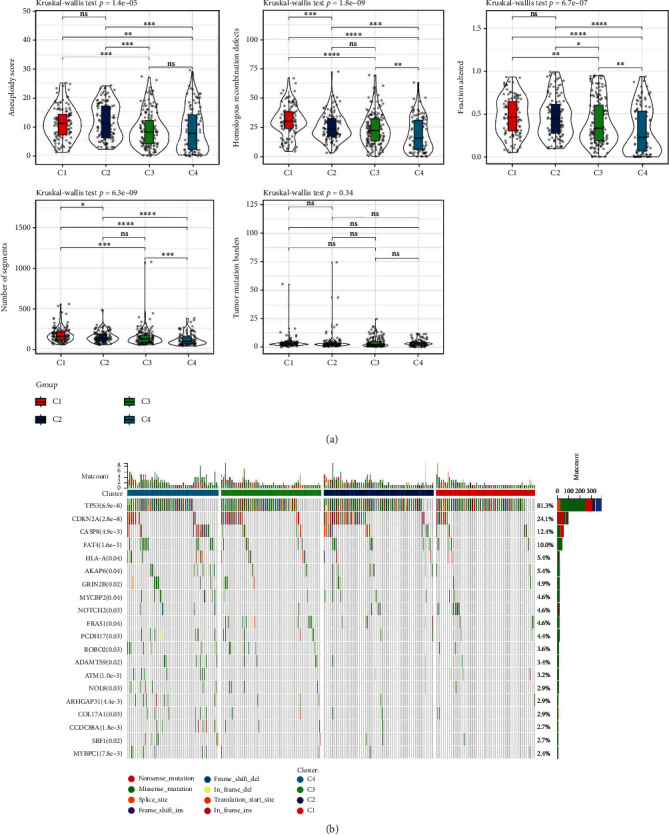
Genomic alterations in the molecular subtypes of the TCGA cohort. (a) Comparison of tumor mutation burden differences, aneuploidy score, homologous recombination defects, number of segments, and fraction altered. (b) Somatic mutations in the four molecular subtypes (chi-square test). ^∗^*P* < 0.05, ^∗∗^*P* < 0.01, ^∗∗∗^*P* < 0.001, and ^∗∗∗∗^*P* < 0.0001.

We explored the differences of genomic alterations in the TCGA cohort (acquired from previous research, [33]) among the four molecular subtypes. C1 subtype showed higher score of aneuploidy, homologous recombination defects, number of segments, and fraction altered (Figure 2(a)). However, no significant difference was shown in tumor mutation burden. In addition, we also analyzed the frequency of gene mutations among molecular subtypes (Figure 2(b)). TP53 had the highest mutation frequency of 81.3% and over a half samples had TP53 mutation. Missense mutation contributed the majority of gene mutation, while nonsense mutation was the most in CDKN2A.

Next, we analyzed whether differential enriched pathways exist in the different molecular subtypes by GSEA. 37 pathways were identified to be significantly enriched in the C1 subtype in the TCGA cohort. The enriched pathways mainly include cancer-related pathways, such as small cell lung cancer, ECM receptor interaction, and Wnt signaling pathway (Figure S2). The results suggested that cellular senescence-associated genes were possibly involved in cancer-related pathways and inflammatory pathways.

### 3.3. Immunological Characteristics and Immunotherapy/Chemotherapy Responses in Different Molecular Subtypes

**Figure 3:**
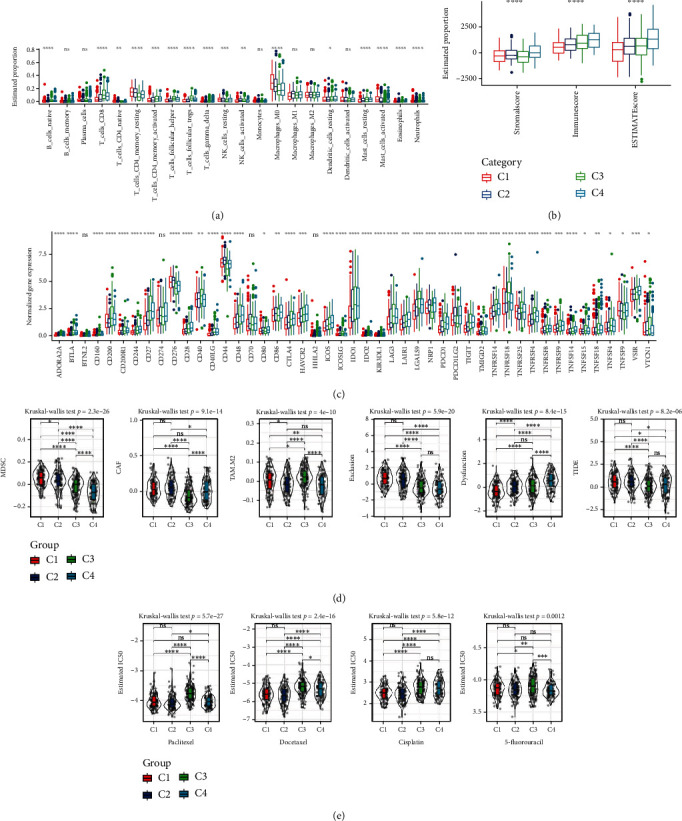
Immune characteristics and immunotherapy/chemotherapy differences between molecular subtypes. (a) The TCGA cohort differences in 22 immune cell scores between molecular subtypes. (b) Differences in ESTIMATE immune infiltration between molecular subtypes in the TCGA cohort. (c) Differentially expressed immune checkpoints between subtypes in the TCGA cohort. (d) Differences in TIDE analysis results between different subgroups in the TCGA cohort. (e) The box plots of the estimated IC50 for paclitaxel, docetaxel, cisplatin, and 5-fluorouracil in the TCGA cohort.

We used immune cell signatures to assess immune cell infiltration in different subtypes to evaluate their immune characteristics. CIBERSORT revealed that 16 of 22 immune cells had a significant difference among four subtypes, such as regulatory T cells, CD8 T cells, resting memory CD4 T cells, and M0 macrophages (*P* < 0.05, Figure 3(a)). C1 had the lowest stromal score and immune score, while C4 had the highest scores (*P* < 0.0001, Figure 3(b)), indicating higher immune infiltration in C4. We considered that differential immune characteristics of four subtypes may result in different immune responses to immunotherapy.

Therefore, we assessed the expression of immune checkpoint genes in different molecular subtypes. We could see that the majority immune checkpoints were differentially expressed among four subtypes (Figure 3(c)), suggesting that the different subtypes may differentially response to immune checkpoint blockade. Not surprisingly, TIDE analysis revealed different responses of four subtypes to immune checkpoint inhibitors. As shown in Figure 3(d), the highest TIDE score was shown in C1, indicating that C1 was more probably to escape from immunotherapy. A higher proportion of MDSCs and CAFs may result in a higher T cell exclusion and unsatisfied immune response (Figure 3(d)). In addition, we also analyzed the predicted response of different molecular subtypes to four chemotherapeutic drugs (paclitaxel, docetaxel, cisplatin, and 5-fluorouracil). C1 and C2 subtypes are more sensitive to paclitaxel, docetaxel, and cisplatin drugs (Figure 3(e)).

### 3.4. Identification of Key Cellular Senescence-Associated Genes

**Figure 4:**
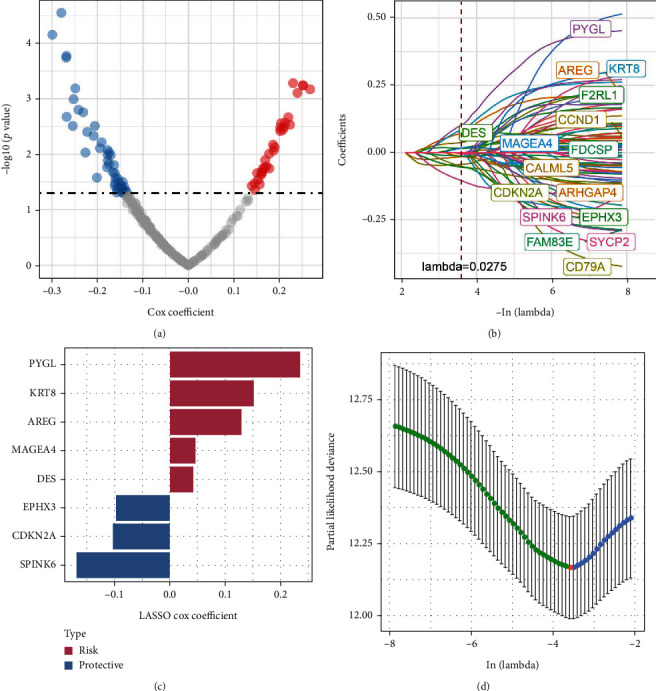
Identification of key cellular senescence-associated genes. (a) A total of 77 promising candidates were identified among the DEGs. (b) Trajectory of each independent variable with lambda. (c) Confidence interval under lambda. (d) LASSO coefficient distribution of gene signature correlated with the senescence.

We then used limma package to screen differentially expressed cellular senescence-associated genes between C1 and non-C1, C2 and non-C2, C3 and non-C3, and C4 and non-C4 molecular subtypes based on the conditions of false discovery rate (FDR) < 0.05 and |log2(fold change)| > 1. 232 DEGs were identified by the above intersection. And 77 prognostic genes were confirmed by the univariate Cox regression analysis including 31 “risk” and 46 “protective” genes (Figure 4(a)). Next, LASSO regression was conducted to compress the 77 prognostic genes. As shown in Figure 4(b), the coefficients of the prognostic genes tended to zero as lambda increased, and the optimal model was confirmed when lambda = 0.0275 (Figure 4(c)). StepAIC was further performed to optimize the model with the least number of prognostic genes. Finally, we identified eight key cellular senescence-associated genes related to prognosis (Figure 4(c)), including PYGL, KRT8, AREG, MAGEA4, DES, EPHX3, CDKN2A, and SPINK6.

### 3.5. Validation of the Eight-Gene Prognostic Model

**Figure 5:**
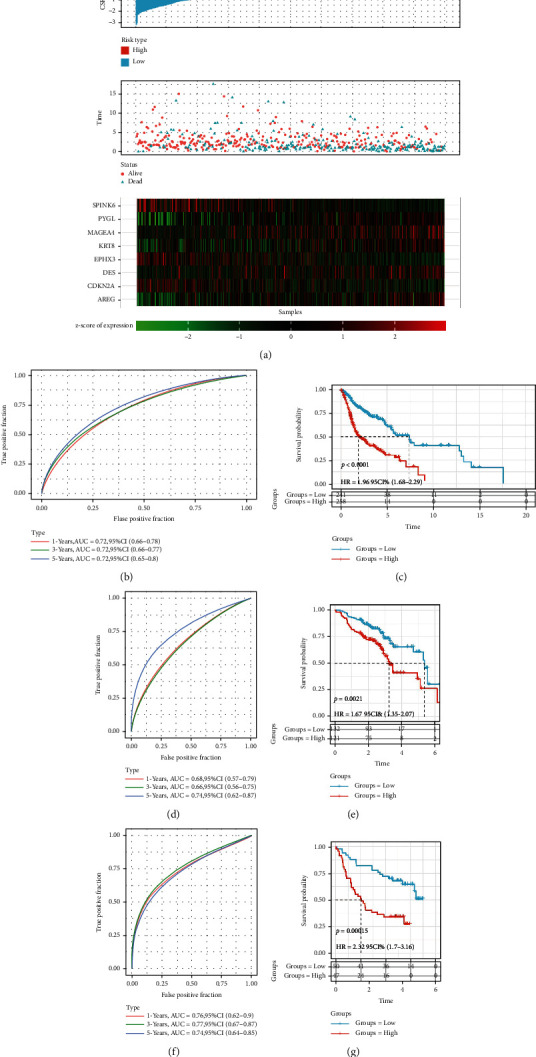
Clinical prognostic modeling and validation. (a) The distribution of survival status, CSRS.Score, and survival time corresponding to senescence-related genes expression in the TCGA cohort. (b) ROC curve with AUC for CSRS.Score classification in the TCGA cohort. (c) KM survival curves of two risk groups in the TCGA cohort. (d, e) ROC curves and KM survival curves of CSRS.Score in the GSE65858 cohort. (f, g) ROC curves and KM survival curves of CSRS.Score in the GSE41613 cohort.

We calculated and normalized the cellular senescence-related signature score (CSRS.Score) for each sample according to the eight-gene model. We classified the samples as the high-risk group if the score was greater than 0 and as the low-risk group if the score was less than 0. The distribution of CSRS.Score for samples in the training set (TCGA cohort) is shown in Figure 5(a). The high-risk group had an obviously higher proportion of dead samples and had a significantly short overall survival. We analyzed the prognostic predictive classification efficiency for 1 year (AUC = 0.72), 3 years (AUC = 0.72), and 5 years (AUC = 0.72), as shown in Figure 5(b). The KM survival curve showed that the overall survival of two risk groups was significantly different (*P* < 0.0001), and higher CSRS.Score had worse overall survival in the training cohort (Figure 5(c)). We further validated the robustness of the eight-gene model in two validation cohorts (GSE65858 and GSE41613), and the similar results were observed (Figures 5(d)–5(g)). For the performance of CSRS.Score in different clinical features, samples were also clearly divided into two risk groups with differential overall survival (*P* < 0.0001, Figure S3).

### 3.6. CSRS.Score Was Correlated with Immune Infiltration and Immunotherapy/Chemotherapy Responses

**Figure 6:**
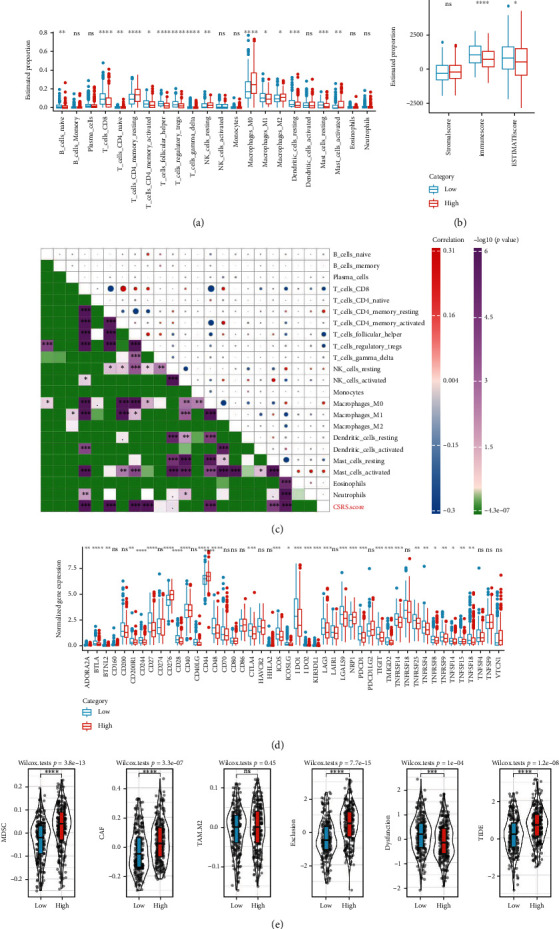
Immune characteristics between two risk groups in the TCGA cohort. (a) The quantity of 22 immune cells analyzed by CIBERSORT. (b) The TCGA cohort immune score and stromal score evaluated by ESTIMATE software. (c) Correlation analysis on CSRS. Score and 22 immune cells in the TCGA cohort. (d) The level of immune checkpoint genes in two risk groups. (e) TIDE analysis for predicting immune response to immune checkpoint blockade. ns: no significance. ^∗∗∗∗^*P* < 0.0001, ^∗∗∗^*P* < 0.001, ^∗∗^*P* < 0.01, and ^∗^*P* < 0.05.

To further explore the immune characteristics between two risk groups, we analyzed the estimated proportion of 22 immune cells in high- and low-risk groups in the TCGA cohort (Figure 6(a)). Some immune cells were differently enriched in high- and low-CSRS.Score subgroups, such as M1 macrophages, activated CD4 memory T cells, and CD8 T cells. Overall, the high-risk group had lower immune infiltration than the low-risk group according to ESTIMATE analysis (Figure 6(b)). The correlation analysis between CSRS.Score and 22 immune cells demonstrated that CSRS.Score was significantly correlated with resting CD4 memory T cells, M0 macrophages, mast cells, CD8 T cells, follicular helper T cells, and regulatory T cells (*P* < 0.001, Figure 6(c)).

We further explored the immune response of two risk groups to immunotherapy. The expression of most immune checkpoints was differential between high- and low-risk groups (Figure 6(d)). TIDE prediction showed that the high-risk group had higher scores of two immunosuppressive cells (MDSC and CAF) probably contributing to higher T cell exclusion score and TIDE score (Figure 6(e) and Figure S4A-B), indicating that the high-risk group was more liable to escape from immunotherapy. In addition to immunotherapeutic response, we also assessed the response of two risk groups to chemotherapeutic drugs (paclitaxel, docetaxel, cisplatin, and 5-fluorouracil). The results showed that the high-risk group was more sensitive to paclitaxel, docetaxel, cisplatin, and 5-fluorouracil (Figure S4C-E).

### 3.7. CSRS.Score Incorporates Clinicopathological Features to Further Improve Survival Prediction

**Figure 7:**
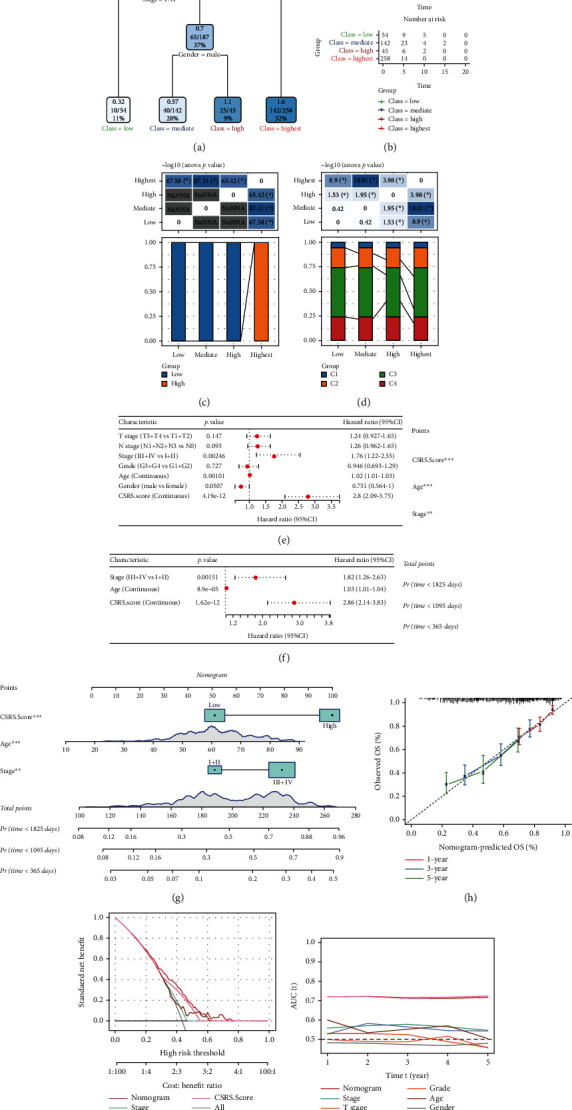
CSRS.Score combined with clinicopathological characteristics to further improve prognostic models and survival prediction. (a) Full-scale annotations of patients including gender, CSRS.Score, age, grade, and TNM stage were applied to develop a survival decision tree for optimizing the risk stratification. (b) Among the four subgroups, significant differences in overall survival could be found. (c, d) Comparative analysis between different subgroups. (e, f) Univariate (e) and multivariate (f) Cox analysis of CSRS.Score and clinicopathological features. (g) A nomogram was developed based on age, CSRS.Score, and stage. (h) Calibration curves for 1, 3, and 5 years for the columnar graph. (i) Decision curve analysis of the nomogram, CSRS.Score, and other clinical features. (j) Compared with other clinicopathological features, the nomogram exhibited the most powerful capacity for survival prediction.

We constructed a decision tree based on clinical information and CSRS.Score, and only stage, gender, and CSRS.Score were remained in the decision tree (Figure 7(a)). Four subgroups including low, median, high, and highest were determined with differential overall survival (Figure 7(b)). Only highest subgroup consisted of high-risk samples (Figure 7(c)) and highest subgroup had the most proportions of C1 and C2 subtypes (Figure 7(d)). Univariate and multifactorial Cox regression analyses illustrated that CSRS.Score was the most significant prognostic factor and age and stage were also independent risk factors (Figures 7(e) and 7(f)). Then, we established a nomogram based on the three independent risk factors for predicting 1-year, 3-year, and 5-year survival (Figure 7(g)). The predictive accuracy of the nomogram was validated by calibration curve (Figure 7(h)). The predicted overall survival of 1 year, 3 years, and 5 years fitted with the observed overall survival, indicating the reliability of the nomogram. Decision curve analysis showed that the nomogram reached relatively high net benefit compared with others (Figure 7(i)). Both nomogram and CSRS.Score had a higher AUC than other clinical features (Figure 7(j)), proving a favorable performance for predicting prognosis in HNSCC patients.

## 4. Discussion

A number of evidences prove that senescence-dependent changes are associated with proinflammatory properties and are involved in the chronic inflammatory microenvironment. Increased levels of inflammation and immune cell infiltration with senescence-dependent changes may lead to tumor formation and malignancy, and their function in HNC is unknown [34]. Exploring the molecular mechanisms of cellular senescence-associated genes is important to determine the role of senescence-dependent changes in HNC. To date, few studies have systematically investigated the molecular mechanisms of cellular senescence-associated genes in HNC and the association between cellular senescence-associated genes and HNC prognosis. We used cellular senescence-associated genes to identify four molecular subtypes by consensus clustering, and these four molecular subtypes differed significantly in prognosis and several clinical features.

Senescence and tumor microenvironment are closely related in tumor progression and invasion. There is an obvious difference of immunity between elder patients and younger patients, where younger patients have more abundant T cells in tumor tissue than elder patients. Senescence leads to a declining immune system which is referred to as immune senescence [35]. It is suggested that tumor-infiltrating CD4+ and CD8+ T cells are reduced in old mice compared to young mice and old mice are more infiltrated with neutrophils and macrophages [36]. Our results showed that CD8 T cells and activated memory CD4 T cells were lower enriched in C1 subtype, while M0 macrophages were extremely higher enriched. C1 also performed a lower stromal and immune infiltration than other subtypes. GSEA results revealed that the immune-related pathways were suppressed in the C1 subtype, which was consistent with its immune features. Therefore, we inferred that cellular senescence-associated genes may play a large role in immune-related pathways and tumor infiltration.

Based on the DEGs among different subtypes, we confirmed a total of eight key cellular senescence prognosis-related genes including CDKN2A, PYGL, KRT8, AREG, MAGEA4, DES, EPHX3, and SPINK6. CDKN2A has been shown to mediate the antitumor effects in HNSCC through cell cycle arrest [37]. Low CDKN2A expression predicts unfavorable prognosis in HPV-negative HNSCC independent of other clinical factors [38], which is accordant with our result that CDKN2A is lower expressed in high-risk group. PYGL is significantly associated with overall survival in HNC patients and may be an independent risk factor for HNSCC prognosis [39]. Keratin 8 (KRT8) overexpression enhanced cell proliferation and migration in gastric cancer and lung cancer, while its decreased expression markedly inhibited cell proliferation, migration, and EMT process [40, 41]. A multiscale integrated analysis figured out KRT8 as a pan-cancer early biomarker [42]. Amphiregulin (AREG) is a ligand of epidermal growth factor receptor (EGFR), which is underlined to function in several aspects of cancerogenesis including cancer cell growth, invasion, metastasis, angiogenesis, and resistance to apoptosis [43].

Additionally, the correlation of molecular subtypes with gene mutations was analyzed, and a significant correlation between the two was detected. The common TP53 and CDKN2A were mutated at a high frequency in the four subtypes. We further evaluated the degree of clinical response of conventional chemotherapeutic drugs paclitaxel, docetaxel, cisplatin, and 5-fluorouracil to CSRS.Score subgroups, and the results showed that high-CSRS.Score to paclitaxel, docetaxel, cisplatin, and 5-fluorouracil was more sensitive. The results suggest that different subgroups have different degrees corresponding to different chemotherapeutic drugs, and perhaps our screened aging-related genes can be used as biomarkers of clinical drug treatment response.

Although the prognostic risk model based senescence-associated genes was illustrated to have robust performance in several independent cohorts, this study did not validate the model in the wet experiments. More clinical HNSCC samples should be included to support the reliability and accuracy of the eight prognostic biomarkers in the future.

## 5. Conclusion

In this study, we developed a prognostic risk model with cellular senescence-associated genes that has great potential as a biomarker for HNSCC patients and provides insights into individualized immunotherapy for head and neck cancer patients.

## Data Availability

The datasets analyzed in this study could be found in GSE65858 at https://www.ncbi.nlm.nih.gov/geo/query/acc.cgi?acc=GSE65858 and GSE41613 at https://www.ncbi.nlm.nih.gov/geo/query/acc.cgi?acc=GSE41613.

## Supplementary Materials

Supplementary Materials Figure S1: clinicopathological characteristics between molecular subtypes. (A–I) Clinicopathological characteristics of molecular subtypes in the TCGA-HNSC cohort, where the lower half is the proportion and the upper half is the statistical significance of the difference in distribution between the two-log10 (*P* value). Figure S2: results of GSEA analysis of pathways between different molecular subtypes in the TCGA cohort. Figure S3: KM curves between high- and low-risk groups of CSRS.Score in different clinical features in the TCGA cohort. Figure S4: response differences to immunotherapy/chemotherapy in two risk groups. (A, B) TIDE analysis results between two risk groups in the GSE65858 (A) and GSE41613 (B) cohorts. (C–E) The box plots of the estimated IC50 for paclitaxel, docetaxel, cisplatin, and 5-fluorouracil in the TCGA cohort, GSE65858, and GSE41613 cohorts. Table S1: a list of 28 prognostic cellular senescence-associated genes used for molecular subtyping.
