# Supplementary material for: Identification and Analysis of Senescence-Related Genes in Head and Neck Squamous Cell Carcinoma by a Comprehensive Bioinformatics Approach
Source: Mediators Inflamm. 2022 Oct 17;2022:4007469. doi: 10.1155/2022/4007469 (PMC9592240; doi:10.1155/2022/4007469)
Supplement: Supplementary Materials — Figure S1: clinicopathological characteristics between molecular subtypes. (A–I) Clinicopathological characteristics of molecular subtypes in the TCGA-HNSC cohort, where the lower half is the proportion and the upper half is the statistical significance of the difference in distribution between the two-log10 (P value). Figure S2: results of GSEA analysis of pathways between different molecular subtypes in the TCGA cohort. Figure S3: KM curves between high- and low-risk groups of CSRS.Score in different clinical features in the TCGA cohort. Figure S4: response differences to immunotherapy/chemotherapy in two risk groups. (A, B) TIDE analysis results between two risk groups in the GSE65858 (A) and GSE41613 (B) cohorts. (C–E) The box plots of the estimated IC50 for paclitaxel, docetaxel, cisplatin, and 5-fluorouracil in the TCGA cohort, GSE65858, and GSE41613 cohorts. Table S1: a list of 28 prognostic cellular senescence-associated genes used for molecular subtyping. [file 4007469.f1.zip › Figure S3.pdf]

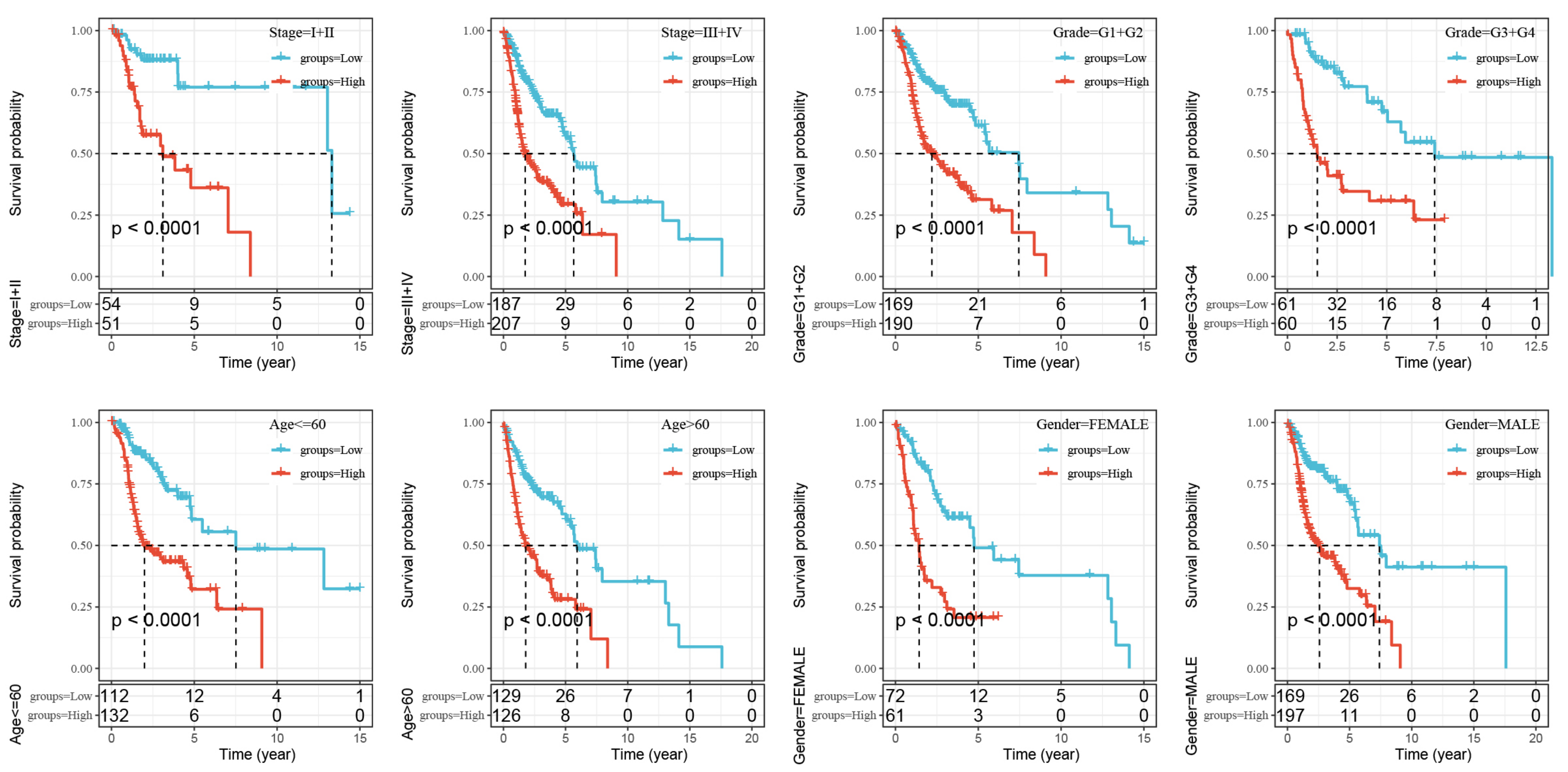
Stage=I+II
groups=Low
groups=High
p < 0.0001
Survival probability
Time (year)
groups=Low 54 9 5 0
groups=High 51 5 0 0
Stage=III+IV
groups=Low
groups=High
p < 0.0001
Survival probability
Time (year)
groups=Low 187 29 6 2 0
groups=High 207 9 0 0 0
Grade=G1+G2
groups=Low
groups=High
p < 0.0001
Survival probability
Time (year)
groups=Low 169 21 6 1
groups=High 190 7 0 0
Grade=G3+G4
groups=Low
groups=High
p < 0.0001
Survival probability
Time (year)
groups=Low 61 32 16 8 4 1
groups=High 60 15 7 1 0 0
Age<=60
groups=Low
groups=High
p < 0.0001
Survival probability
Time (year)
groups=Low 112 12 4 1
groups=High 132 6 0 0
Age>60
groups=Low
groups=High
p < 0.0001
Survival probability
Time (year)
groups=Low 129 26 7 1 0
groups=High 126 8 0 0 0
Gender=FEMALE
groups=Low
groups=High
p < 0.0001
Survival probability
Time (year)
groups=Low 72 12 5 0
groups=High 61 3 0 0
Gender=MALE
groups=Low
groups=High
p < 0.0001
Survival probability
Time (year)
groups=Low 169 26 6 2 0
groups=High 197 11 0 0 0
