# Supplementary figures and images for: Identification and Analysis of Senescence-Related Genes in Head and Neck Squamous Cell Carcinoma by a Comprehensive Bioinformatics Approach
Source: Mediators Inflamm. 2022 Oct 17;2022:4007469. doi: 10.1155/2022/4007469 (PMC9592240; doi:10.1155/2022/4007469)

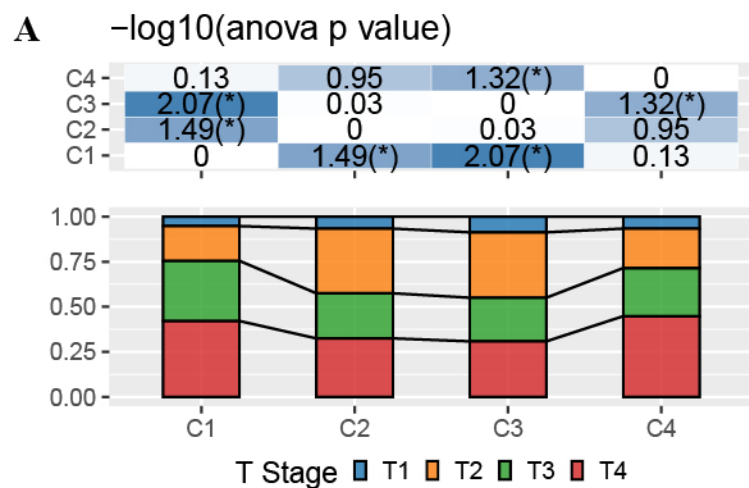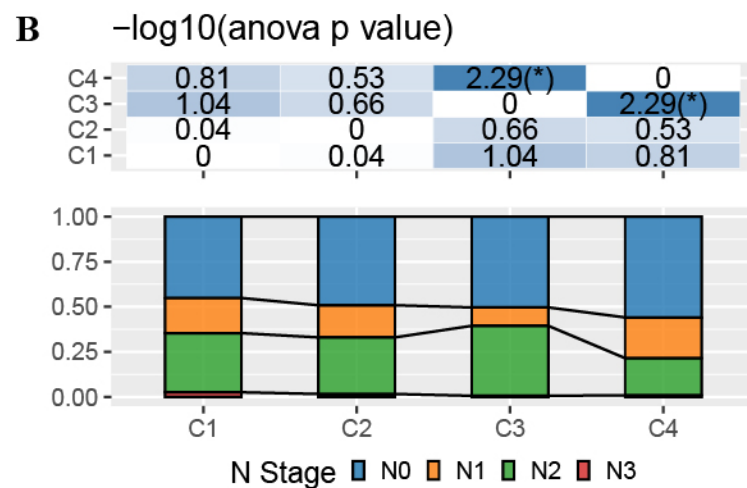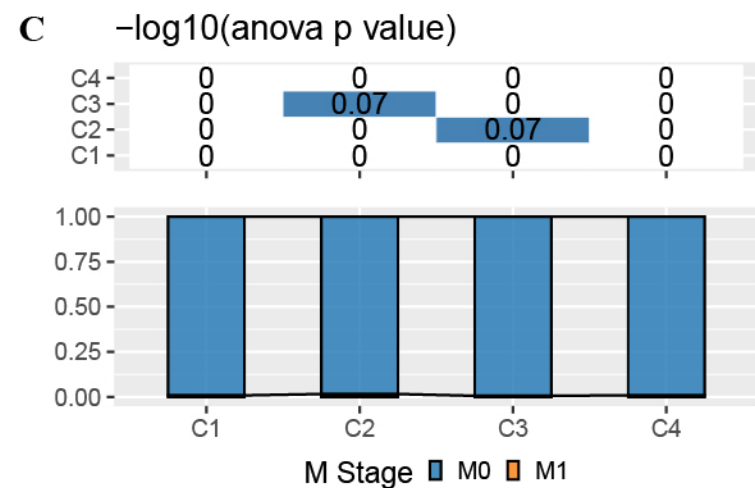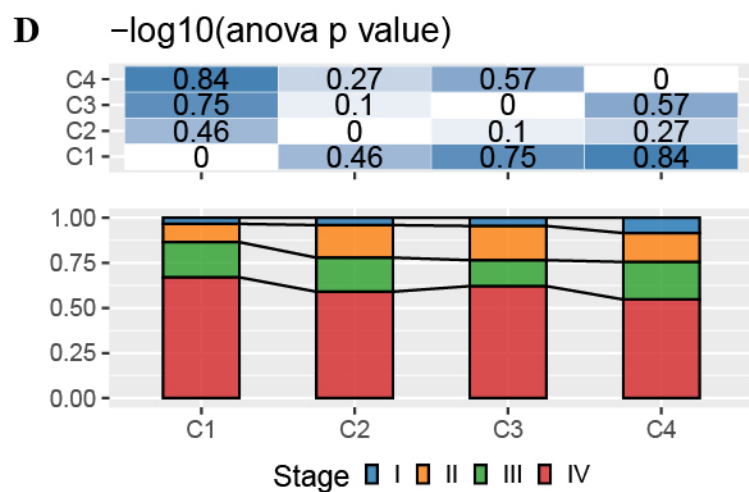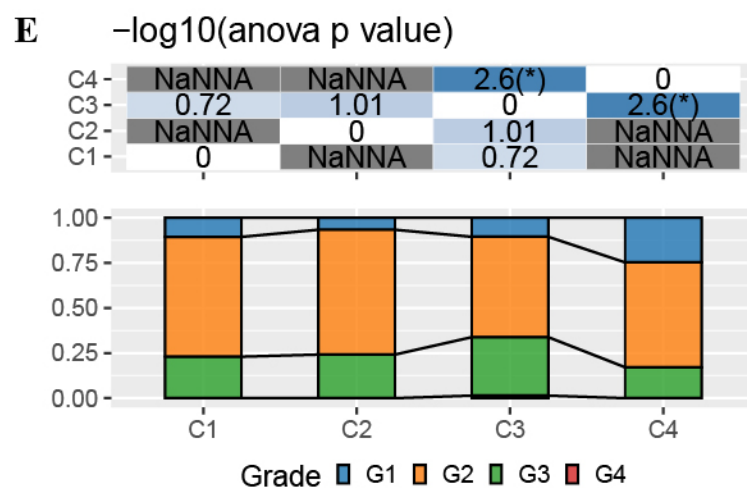

Supplement: Supplementary Materials — Figure S1: clinicopathological characteristics between molecular subtypes. (A–I) Clinicopathological characteristics of molecular subtypes in the TCGA-HNSC cohort, where the lower half is the proportion and the upper half is the statistical significance of the difference in distribution between the two-log10 (P value). Figure S2: results of GSEA analysis of pathways between different molecular subtypes in the TCGA cohort. Figure S3: KM curves between high- and low-risk groups of CSRS.Score in different clinical features in the TCGA cohort. Figure S4: response differences to immunotherapy/chemotherapy in two risk groups. (A, B) TIDE analysis results between two risk groups in the GSE65858 (A) and GSE41613 (B) cohorts. (C–E) The box plots of the estimated IC50 for paclitaxel, docetaxel, cisplatin, and 5-fluorouracil in the TCGA cohort, GSE65858, and GSE41613 cohorts. Table S1: a list of 28 prognostic cellular senescence-associated genes used for molecular subtyping. [file 4007469.f1.zip › Figure S1.pdf]

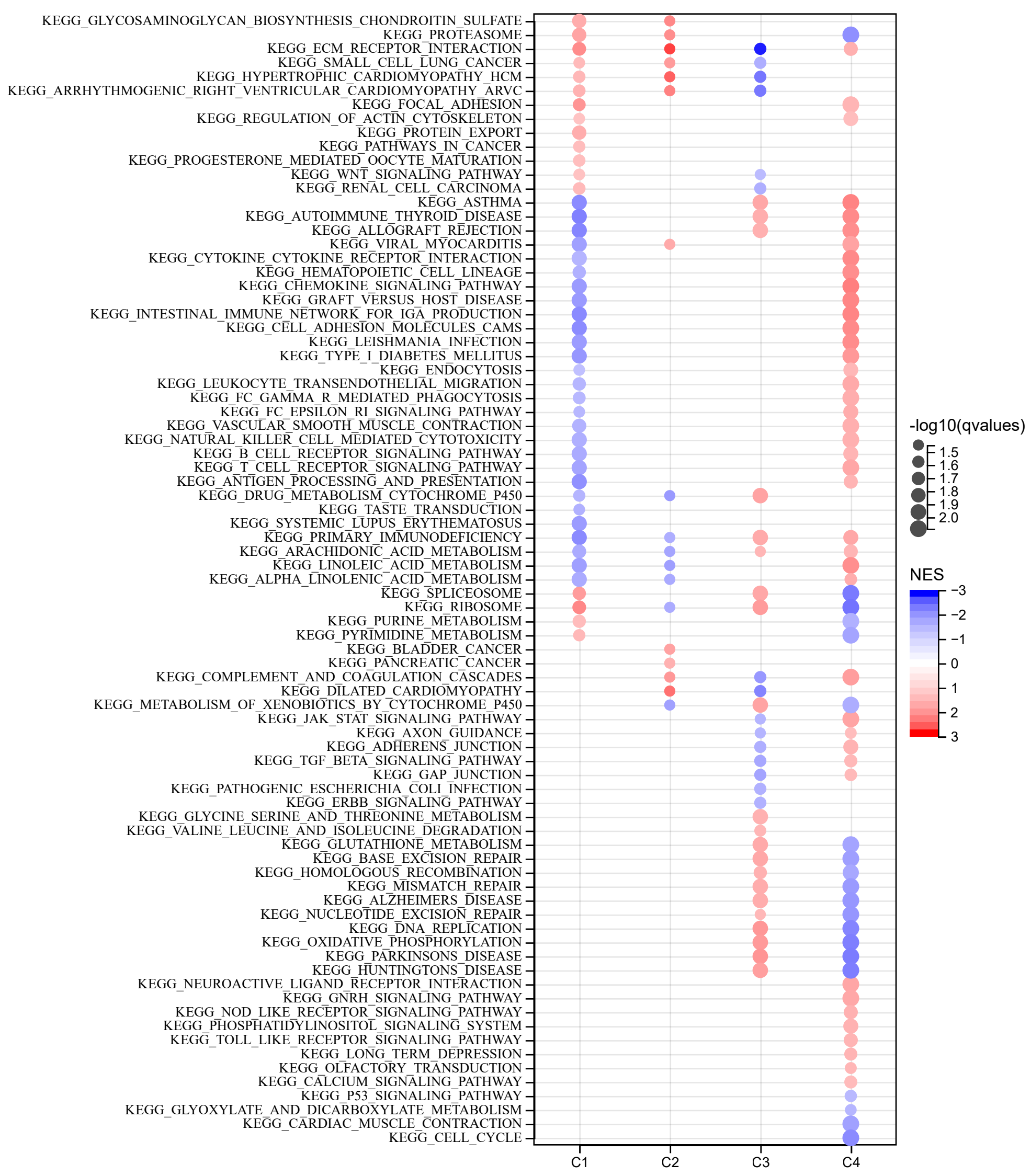

Supplement: Supplementary Materials — Figure S1: clinicopathological characteristics between molecular subtypes. (A–I) Clinicopathological characteristics of molecular subtypes in the TCGA-HNSC cohort, where the lower half is the proportion and the upper half is the statistical significance of the difference in distribution between the two-log10 (P value). Figure S2: results of GSEA analysis of pathways between different molecular subtypes in the TCGA cohort. Figure S3: KM curves between high- and low-risk groups of CSRS.Score in different clinical features in the TCGA cohort. Figure S4: response differences to immunotherapy/chemotherapy in two risk groups. (A, B) TIDE analysis results between two risk groups in the GSE65858 (A) and GSE41613 (B) cohorts. (C–E) The box plots of the estimated IC50 for paclitaxel, docetaxel, cisplatin, and 5-fluorouracil in the TCGA cohort, GSE65858, and GSE41613 cohorts. Table S1: a list of 28 prognostic cellular senescence-associated genes used for molecular subtyping. [file 4007469.f1.zip › Figure S2.pdf]

**A**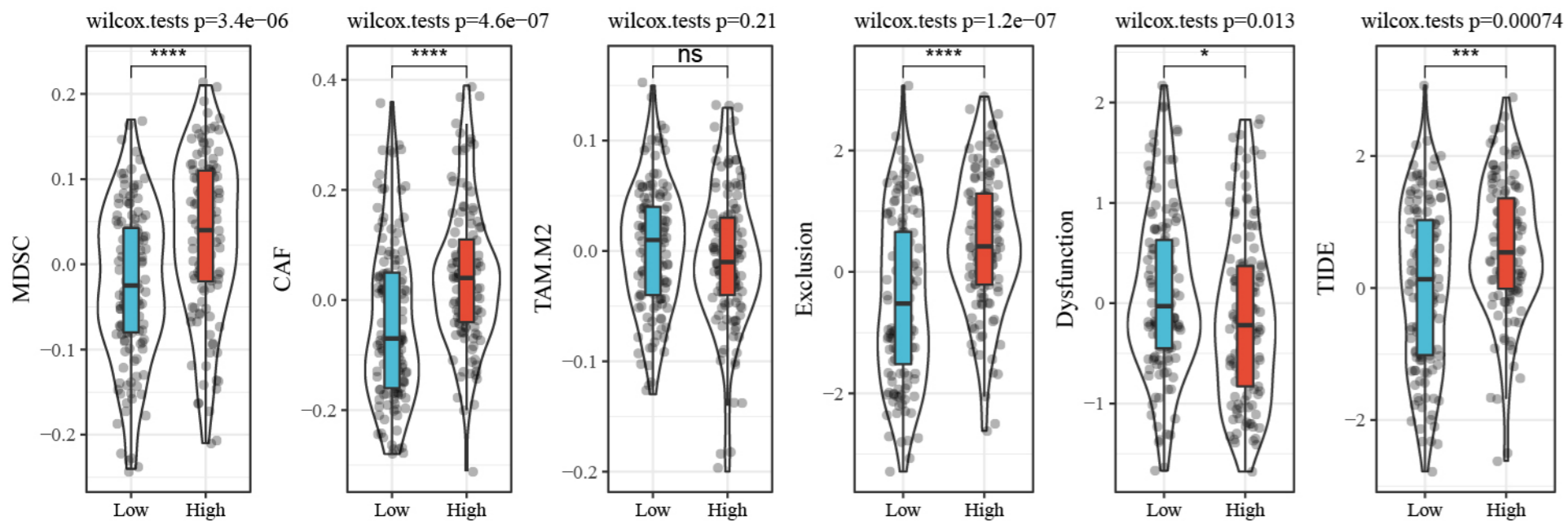**B**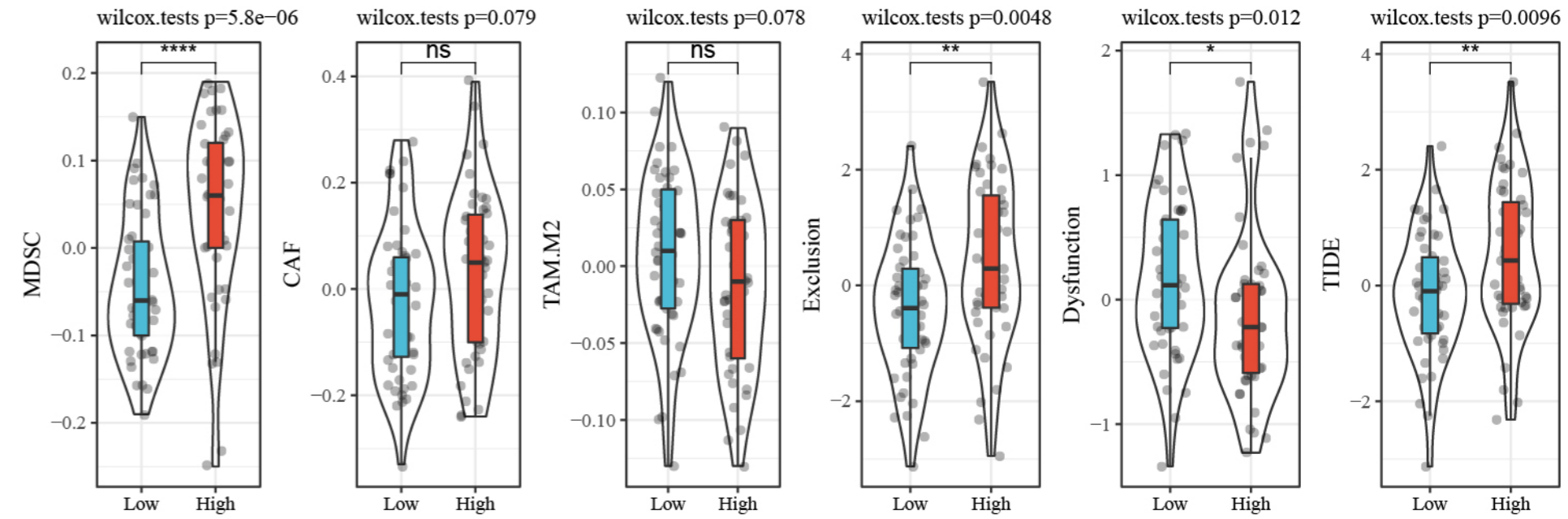**C**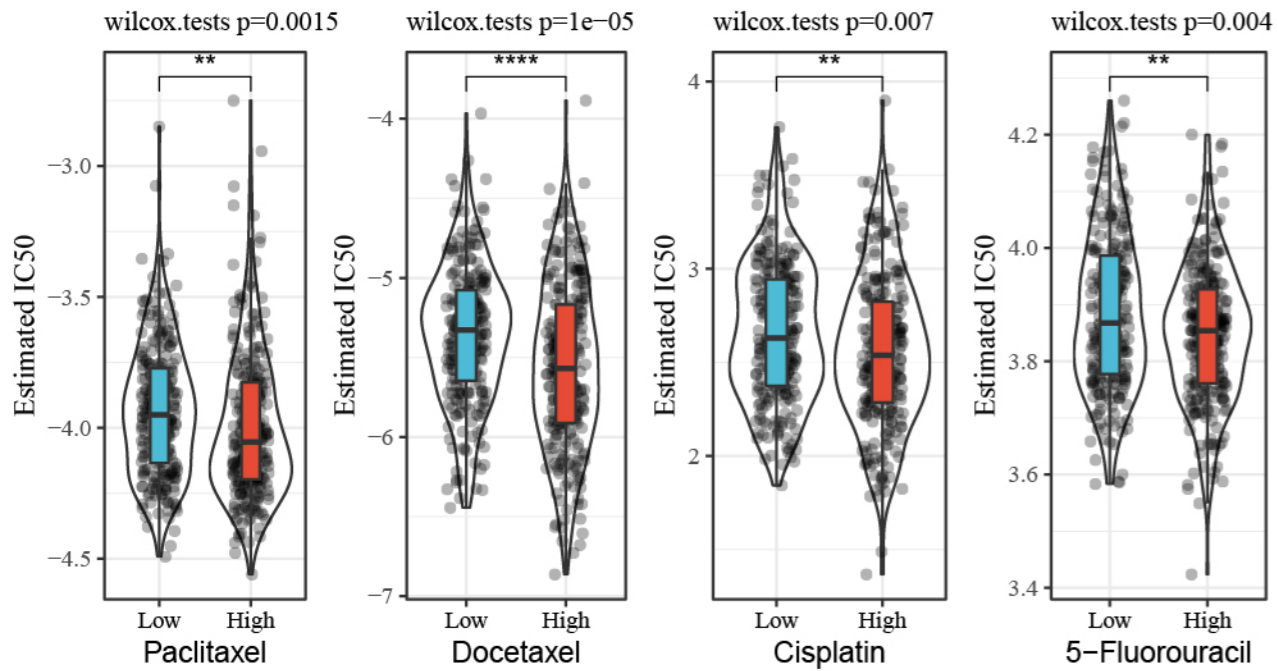**D**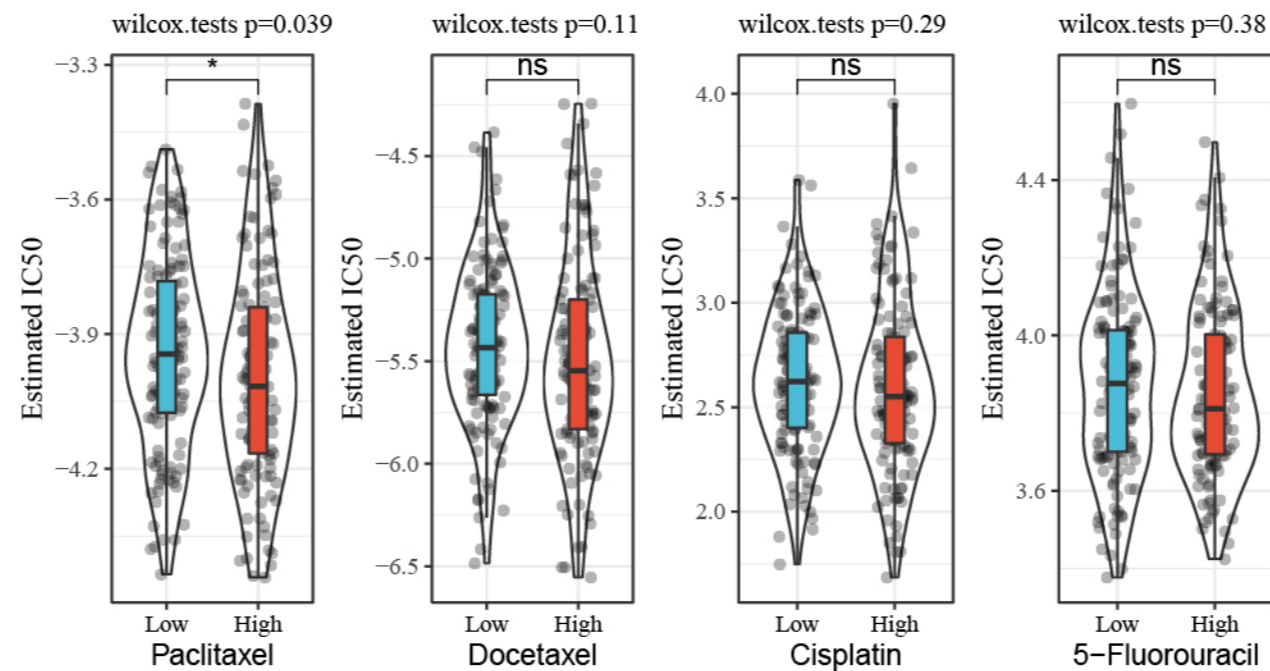**E**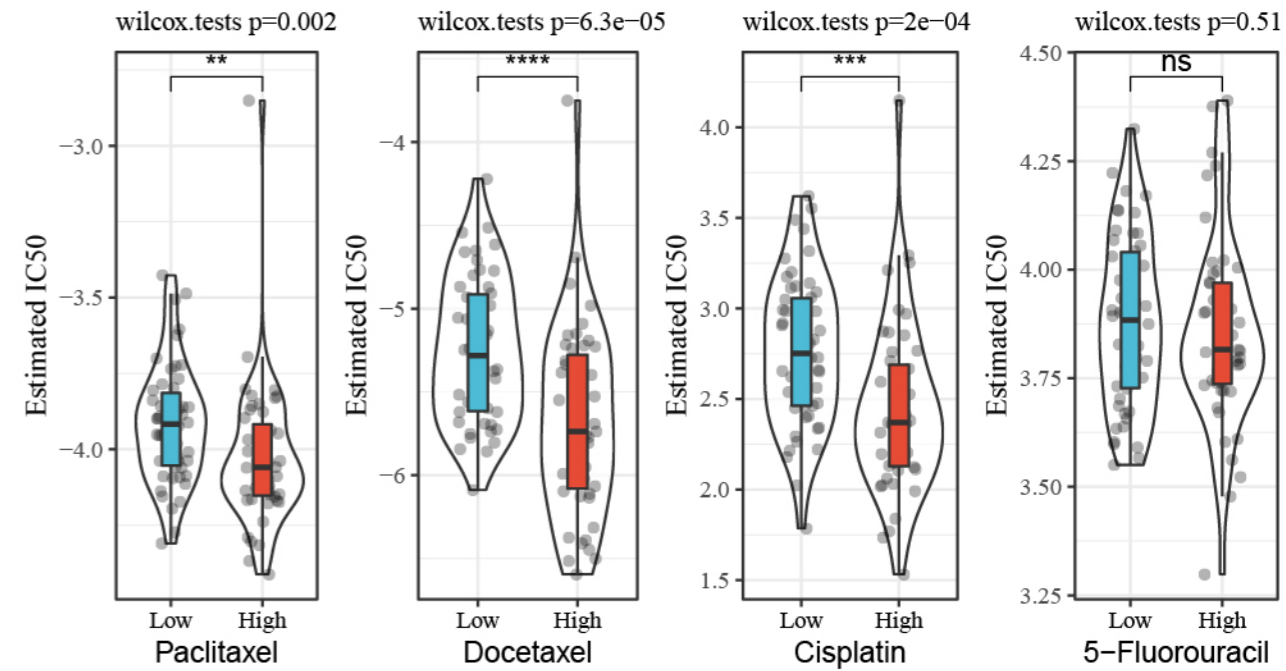

Supplement: Supplementary Materials — Figure S1: clinicopathological characteristics between molecular subtypes. (A–I) Clinicopathological characteristics of molecular subtypes in the TCGA-HNSC cohort, where the lower half is the proportion and the upper half is the statistical significance of the difference in distribution between the two-log10 (P value). Figure S2: results of GSEA analysis of pathways between different molecular subtypes in the TCGA cohort. Figure S3: KM curves between high- and low-risk groups of CSRS.Score in different clinical features in the TCGA cohort. Figure S4: response differences to immunotherapy/chemotherapy in two risk groups. (A, B) TIDE analysis results between two risk groups in the GSE65858 (A) and GSE41613 (B) cohorts. (C–E) The box plots of the estimated IC50 for paclitaxel, docetaxel, cisplatin, and 5-fluorouracil in the TCGA cohort, GSE65858, and GSE41613 cohorts. Table S1: a list of 28 prognostic cellular senescence-associated genes used for molecular subtyping. [file 4007469.f1.zip › Figure S4.pdf]
